# Supplementary material for: Watching the release of a photopharmacological drug from tubulin using time-resolved serial crystallography
Source: Nat Commun. 2023 Feb 17;14:903. doi: 10.1038/s41467-023-36481-5 (PMC9936131; doi:10.1038/s41467-023-36481-5)
Supplement: Supplementary file 1 — Supplementary Information [file 41467_2023_36481_MOESM1_ESM.docx]

Supplementary Material for

**Watching the release of a photopharmacological drug from tubulin using time-resolved serial crystallography**

Maximillian Wranik^1^, Tobias Weinert^1^, Chavdar Slavov^2^, Tiziana Masini^3^, Antonia Furrer^1^, Natacha Gaillard^1^, Dario Gioia^3^, Marco Ferrarotti^3^, Daniel James^1^, Hannah Glover^1^, Melissa Carrillo^1^, Demet Kekilli^1^, Robin Stipp^1^, Petr Skopintsev^1^, Steffen Brünle^1^, Tobias Mühlethaler^1^, John Beale^1^, Dardan Gashi^4^, Karol Nass^4^, Dmitry Ozerov^5^, Philip J.M. Johnson^4^, Claudio Cirelli^4^, Camila Bacellar^4^, Markus Braun^2^, Meitian Wang^4^, Florian Dworkowski^4^, Chris Milne^4^, Andrea Cavalli^3,6^, Josef Wachtveitl^2^, Michel O. Steinmetz^1,7,*^, Jörg Standfuss^1,*^

^1^ Division of Biology and Chemistry, Paul Scherrer Institut, 5232 Villigen, Switzerland.

^2^ Institute of Physical and Theoretical Chemistry, Goethe University, Frankfurt am Main, Germany.

^3^ Computational & Chemical Biology, Istituto Italiano di Tecnologia, 16163 Genova, Italy.

^4^ Photon Science Division, Paul Scherrer Institut, 5232 Villigen, Switzerland.

^5^ Scientific Computing, Theory and Data, Paul Scherrer Institut, 5232 Villigen, Switzerland.

^6^ Department of Pharmacy and Biotechnology, University of Bologna, 40126 Bologna, Italy.

^7^ Biozentrum, University of Basel, 4056 Basel, Switzerland.

*Correspondence to: michel.steinmetz@psi.ch and joerg.standfuss@psi.ch

**This PDF file includes:**

Supplementary Figures. S1 to S6

Supplementary Tables S1 to S2


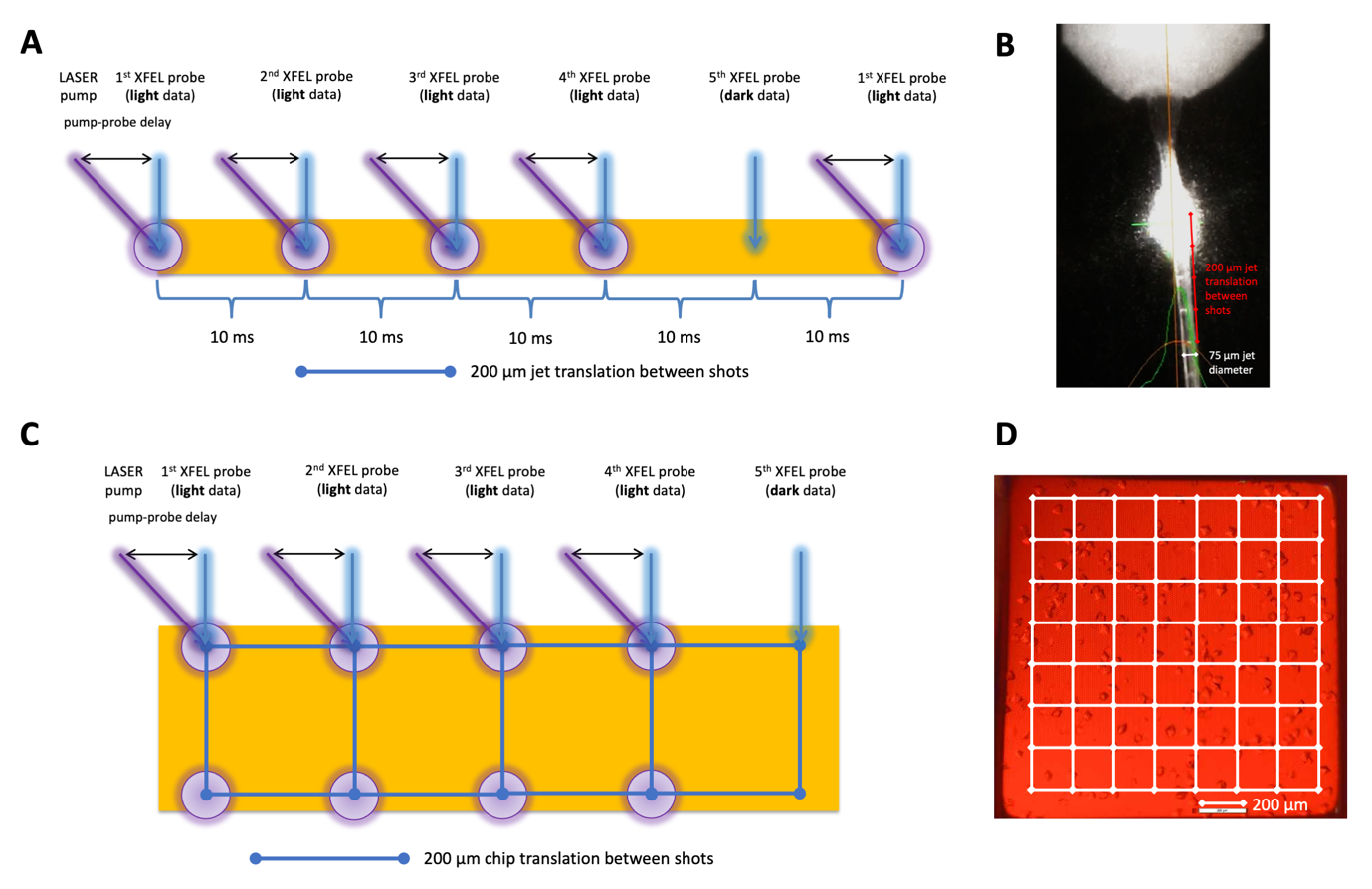


**Supplementary Figure 1: Experimental setup.** (**A**) The 4:1 pump-probe scheme used with the high-viscosity sample extruder^45^ we introduced for crystal delivery time-resolved crystallography experiments^46^ is depicted. Distance between shots taken at 100 Hz was about 200 μm and the laser spot size (1/e^2^) was about 65 μm (depicted as purple circle). Every 5^th^ pattern was taken without the laser turned on. In this setup each time delay was collected in about 50 minutes measuring time and needed approximately 5 mg of tubulin. Notably, we suggest to optimize sample injection before the experiment to increase sample and time efficiency during the beamtime^47^. Alternative sample delivery methods like solid-supports^48^ or tape-drives^49^ could be used to target longer time delays and thus measure ligands with longer residence times. (**B**) Image showing the laser on the jet and the penetration marks of the XFEL. Taking the 75 μm jet thickness to provide a scale in the picture it is evident that individual shots which are visible as white lines on the jet are evenly spaced. The laser glow in the image is overloaded and cannot be used to draw conclusions on the focal spot size of the laser, which was accurately measured using a knife edge scan at the sample position.


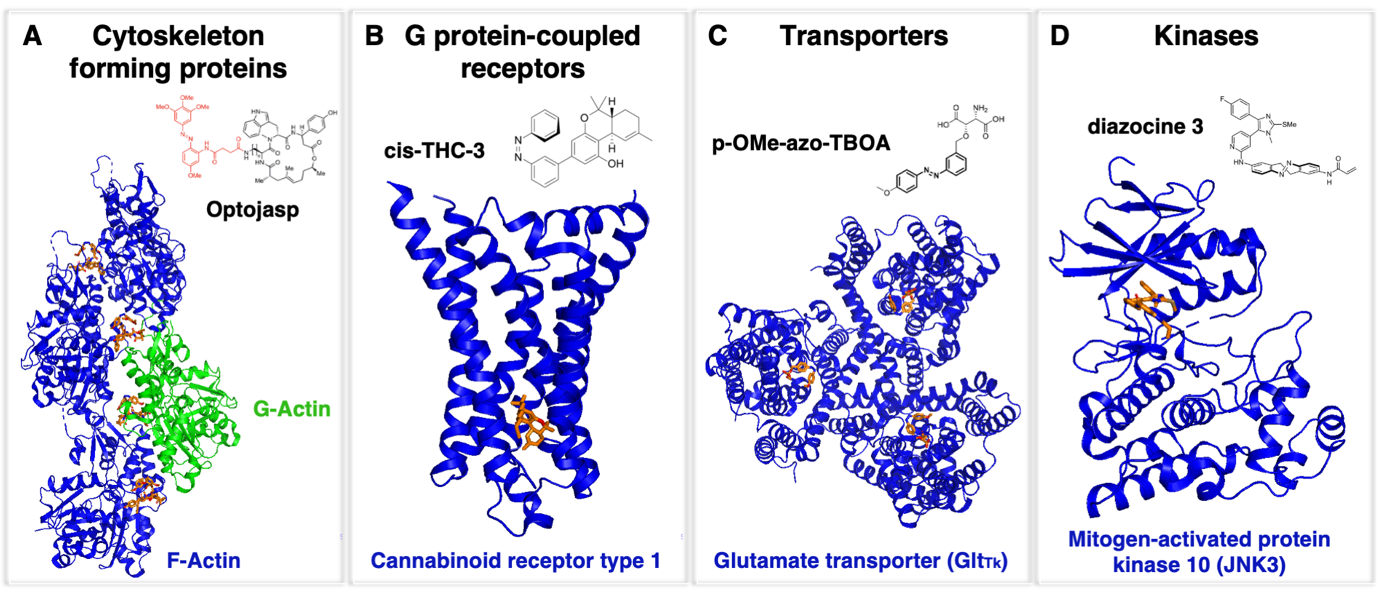


**Supplementary Figure 2: Selection of protein classes that have been targeted with photopharmacological compounds.** (**A**) A class of cytoskeletal proteins: Actin. Most abundant cellular protein and an essential component of the eukaryotic cytoskeleton^50, 51, 52^. (**B**) A class of G protein-coupled receptors: Cannabinoid receptor type 1. One of the most widely expressed GPCR in the central nervous system. Its activation is associated with mood, motor coordination, memory, and recognition^53, 54^. (**C**) A class of membrane transporters: Glutamate transporter Glt_Tk_. Disruption linked to neurotoxicity under ischemic conditions and epilepsy^55^. (**D**) A class of signaling kinases: Mitogen-activated protein kinase 10 (JNK3). A key signaling enzyme in the cellular stress response, targeted for the treatment of neurodegenerative diseases including Alzheimer’s, Huntington’s, and Parkinson’s disease^56^. These examples suggest that the approach to employ photochemical affinity switches to study protein-ligand interaction dynamics could be expanded to a series of important targets from pharmacologically relevant protein families.

**
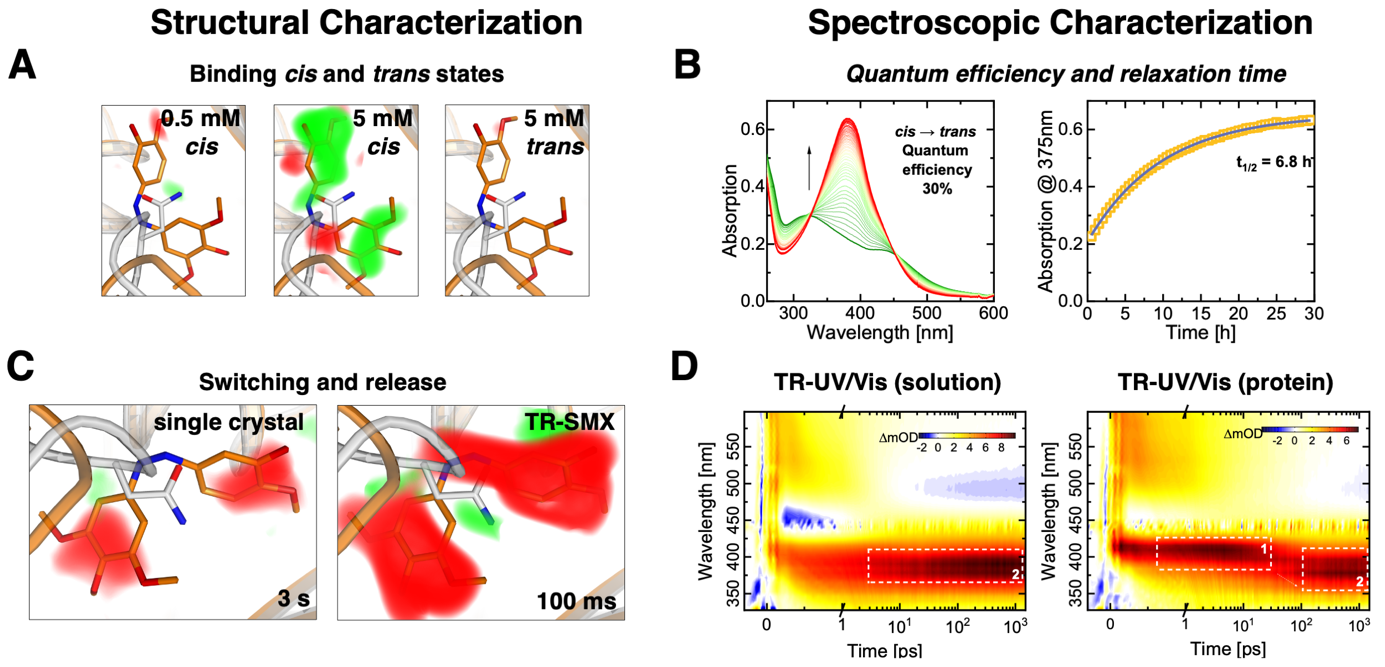
**

**Supplementary Figure 3: Initial characterization of the azo-CA4 photoswitch. (A)** Soaking of tubulin crystals with 0.5 mM *cis*-azo-CA4, 5 mM *cis*-azo-CA4, or 5 mM *trans*-azo-CA4. The binding pocket remained unoccupied up to azo-CA4 concentrations of 5 mM when the ligand was not exposed to light, suggesting that a light-triggered release of azo-CA4 should be feasible. (**B**) Titration experiments used to determine the quantum efficiency of conversion and relaxation of azo-CA4 over time in crystallization buffer. The quantum efficiency of 30% is large enough to reach sufficient activation levels in time-resolved serial crystallography experiments. We further confirmed that the *cis*-to-*trans* thermal relaxation is slow enough to maintain the *cis* conformation without constant illumination during a single sample injection of about 30 minutes. (**C**) Ligand release after photoinduced isomerization followed by a brief annealing of a single cryo-cooled crystal soaked with 1.25 mM of *cis*-azo-CA4, and ligand release after 100 ms in a time-resolved serial crystallography experiment. A single tubulin crystal was soaked with 1.25 mM of *cis*-azoCA4 before collecting a conventional cryo-dataset. Light data was collected by blocking the cryo-stream and illuminating the crystal before collecting a second dataset on a crystal position ~150 μm away from the first position. The isomorphous difference map between both datasets showed that azo-CA4 is released from the crystal upon photoswitching. We then moved on to a time-resolved serial crystallography experiment at the synchrotron and found that the ligand was fully released from its tubulin-binding site after 100 ms of illumination. (**D**) Time-resolved UV-Vis spectroscopy revealed that tubulin modifies the compound relaxation behavior up to the late picosecond range, suggesting that the ligand isomerization inside the binding pocket is finished after 1 ns. All panels show isomorphous difference maps that are displayed in red (negative) and green (positive) at 3 σ. The substrate free apo form of tubulin is shown in grey and the substrate bound form in orange.

**
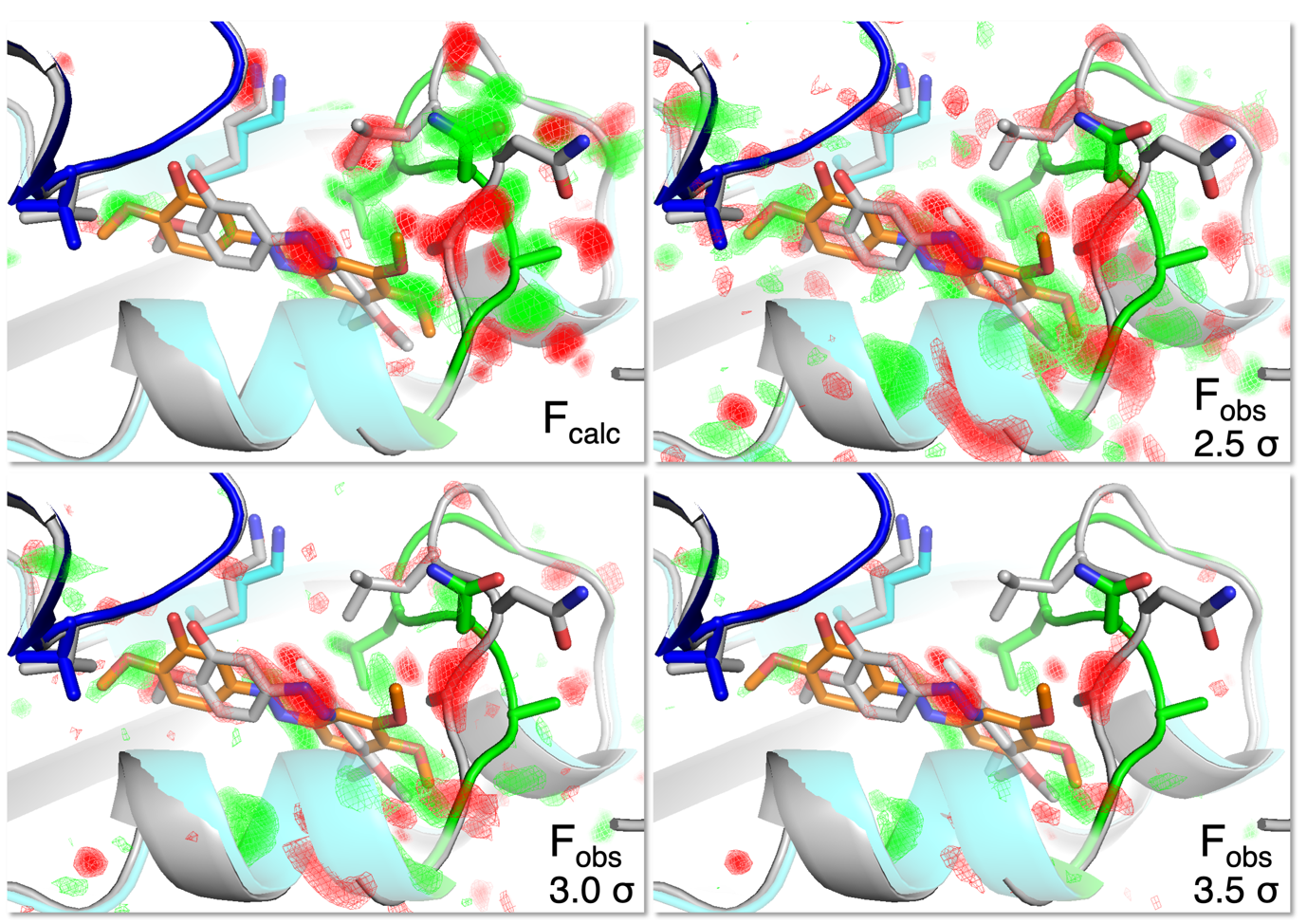
**

**Supplementary Figure 4: Comparison of F_obs_ and F_calc_ difference maps.** Experimentally observed F_obs_(1ms)-F_obs_(dark) and calculated F_calc_(1ms-model)-F_calc_(dark-model) difference density maps within the ligand binding site are shown. Positive and negative difference densities are displayed in green and red, respectively. The initial dark model is shown in grey and the 1 ms structure as blue (α tubulin), cyan (β tubulin), and green (βT7 loop). Selected side chains are displayed in stick representation. The F_calc_ difference density map (top) was calculated using B-factors of 30 for all atoms and is displayed at a sigma level of 3.5 sigma, while the F_obs_ maps are shown at levels from 2.5 to 3.5 sigma. The F_calc_ maps represent a full activation level and data without noise and even though this prevents direct comparison of sigma levels, there is an excellent agreement between the simulated and calculated difference maps.


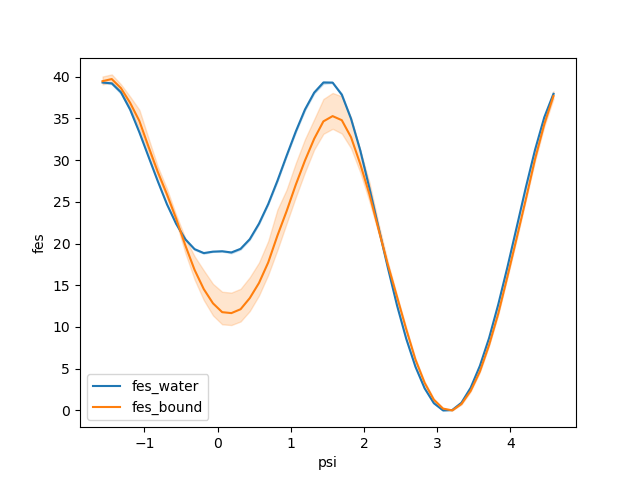


**∆∆G_cis-trans_**

*cis*

*trans*

**Supplementary Figure 5: Computational analysis of the binding energy landscape provides a rationale for how cis-trans isomerization initiates ligand unbinding.** The plot indicates the energy gain of the *cis* vs. the *trans* azo-CA4 isomer upon binding to tubulin. A single WTM simulation was carried out with a custom protocol to sample conformations compatible to the XFEL-based time-resolved structures. Block analysis with block size of 50 ns was performed to assess convergence and to plot 95% confidence interval error bands around converged values.

**
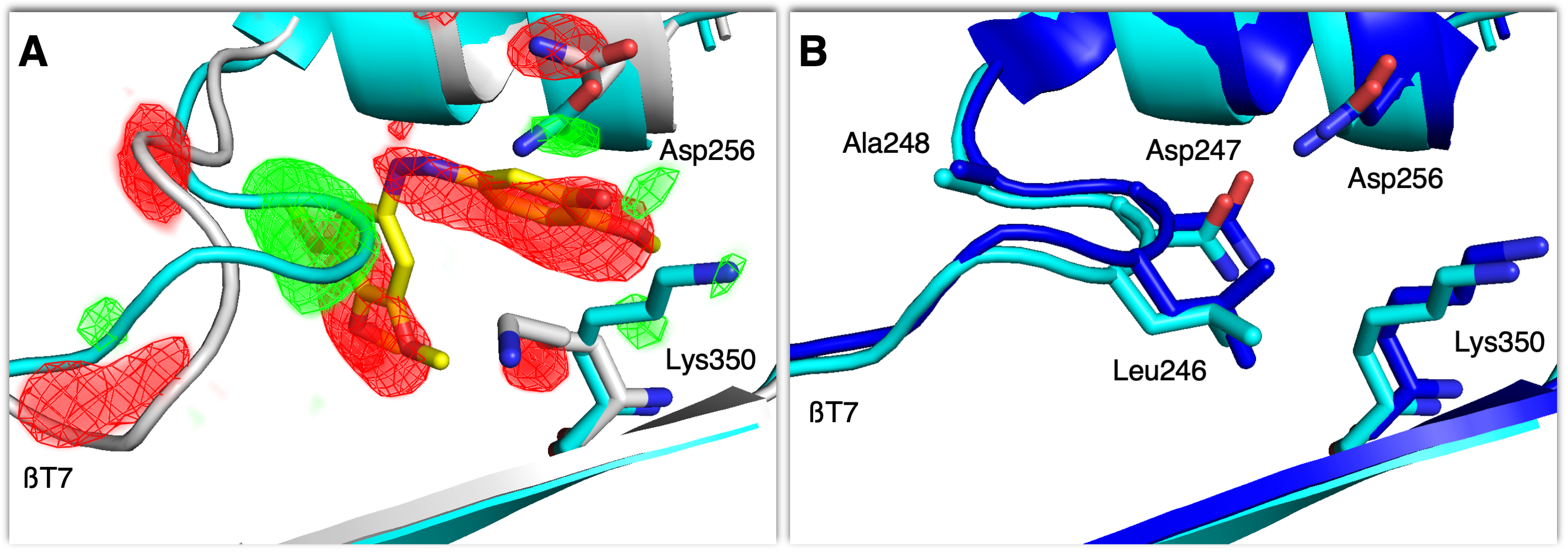
**

**Supplementary Figure 6:** **Comparison of tubulin-azo-CA4 complex after photoinduced ligand release with a structure of apo-tubulin**. (**A**) Overlay of liganded dark (grey) and 100 ms (cyan) tubulin structures obtained by serial synchrotron crystallography. The corresponding difference densities (positive in green, negative in red, F_obs_(100 ms)-F_obs_(dark), sigma level = 5.0) confirm the release of azo-CA4 and reorganization of the colchicine-site in tubulin. (**B**) Overlay of the 100 ms structure (cyan) and the one determined using data from apo-tubulin crystals (blue).

**Supplementary Table 1: Crystallographic data statistics**

|  | **Dark**  **(XFEL)** | **1 ns**  **(XFEL)** | **10 ns**  **(XFEL)** | **100 ns**  **(XFEL)** | **1 ms**  **(XFEL)** | **10 ms**  **(XFEL)** | **100 ms**  **(XFEL)** | **1 ms**  **(XFEL)** | **10 ms**  **(XFEL)** | **Apo**  **(XFEL)** | **100 ms**  **(SYN)** | **Dark**  **(SYN)** |
| --- | --- | --- | --- | --- | --- | --- | --- | --- | --- | --- | --- | --- |
| **Data collection** | | | | | | | | | | | | |
| **Space group** | P2_1_ | | | | | | | | | | | |
| *a*, *b*, *c* (Å) | 74.5, 92.6, 84.0 | | | | | | | | | 74.7, 92.7, 84.1 | 74.3, 91.9, 83.7 | |
| a, b, g  **(**°**)** | 90, 96.7, 90 | | | | | | | | | 90, 96.4, 90 | 90, 96.82, 90 | |
| **Indexed patterns** | 370356 | 63970 | 69058 | 61091 | 73798 | 67496 | 57915 | 60644 | 89399 | 82666 | 41314 | 102238 |
| **Indexing rate (%)** | 16.6 | 23.3 | 18.1 | 19.8 | 13.2 | 23.5 | 31.2 | 16.7 | 22.6 | 25.6 | 18.1 | 16.5 |
| **Overall statistics: 11.09 Å – 1.70 Å**  **(high-resolution statistics: 1.76 Å – 1.70 Å)** | | | | | | | | | | **11.09-1.80 Å**  **(1.86-1.80 Å)** | **92.11-2.1 Å**  **(2.18-2.10 Å)** | |
| **No. reflections** | 123960 (12356) | 122155(12154) | 122150  (12154) | 122150  (12154) | 122150  (12154) | 122154  (12154) | 122150  (12154) | 122153  (12154) | 122152  (12154) | 104994  (10494) | 38945  (117) | 43965  (945) |
| **Completeness (%)** | 100  (100) | 100  (100) | 100  (100) | 100  (100) | 100  (100) | 100  (100) | 100  (100) | 100  (100) | 100  (100) | 100  (100) | 60  (2) | 67  (15) |
| **Multiplicity** | 2089.8  (1000.6) | 365.9  (188.4) | 347.5  (175.9) | 314.1  (164.8) | 404.2  (208.4) | 392.6  (207.5) | 309.1  (155.8) | 328.9  (161.2) | 586.1  (313.1) | 429.5  (194.3) | 274.2  (216.6) | 602.5  (504.6) |
| **R_split_ (%)** | 7.5  (78.85) | 16.9  (217.8) | 17.2  (204.3) | 18.1  (180.4) | 16.6  (268.7) | 16.7  (174.4) | 17.5  (299.0) | 18.1  (253.1) | 13.8  (106.5) | 14.2  (372.3) | 10.5  (69.48) | 8.0  (49.9) |
| ***CC_1/2_*** | 0.992  (0.694) | 0.971  (0.265) | 0.968  (0.274) | 0.964  (0.323) | 0.972  (0.210) | 0.971  (0.343) | 0.969  (0.170) | 0.968  (0.217) | 0.979  (0.558) | 0.981  (0.14) | 0.992  (0.528) | 0.996  (0.660) |
| ***<I/σ(I)>*** | 9.53  (1.43) | 4.01  (0.50) | 4.16  (0.56) | 4.05  (0.62) | 4.05  (0.43) | 4.26  (0.64) | 3.68  (0.38) | 3.79  (0.45) | 5.36  (1.03) | 4.59  (0.32) | 7.87  (1.25) | 10.09  (1.62) |
| **PDB Code** | 7YYQ | 7YYV | 7YYW | 7YYX | 7YYY | 7YYZ | 7YZ0 | 7YZ1 | 7YZ2 | 7YZ3 | 7YZ5 | 7YZ6 |

**Supplementary Table 2: Refinement statistics**

|  | **Dark**  **(XFEL)** | **1 ns**  **(XFEL)** | **10 ns**  **(XFEL)** | **100 ns**  **(XFEL)** | **1 μs**  **(XFEL)** | **10 μs**  **(XFEL)** | **100 μs**  **(XFEL)** | **1 ms**  **(XFEL)** | **10 ms**  **(XFEL)** | **Apo**  **(XFEL)** | **~100 ms**  **(SYN)** | **Dark**  **(SYN)** |
| --- | --- | --- | --- | --- | --- | --- | --- | --- | --- | --- | --- | --- |
| **Resolution (Å)** | 9.5 Å – 1.7 Å | 9.5 Å – 2.2 Å | 9.5 Å – 2.2 Å | 9.5 Å – 2.2 Å | 9.5 Å – 2.2 Å | 9.5 Å – 2.2 Å | 9.5 Å – 2.2 Å | 9.5 Å – 2.2 Å | 9.5 Å – 2.2 Å | 9.5 Å – 1.80 Å | 73.75 Å – 2.1 Å | 73.75 Å – 2.1 Å |
| **No. reflections** | 123591 (8816) | 53409  (3901) | 53221  (3910) | 52461  (3763) | 52234  (3710) | 53180  (3840) | 52580  (3778) | 53111  (3915) | 53134  (3657) | 102745  (1827) | 38921  (68) | 43943  (652) |
| ***R*_work_ / *R*_free_ in %** | 12.13 /  15.38 | 31.11 /  35.19 | 31.13 /  34.33 | 31.10 /  35.53 | 30.46 /  34.88 | 29.87 /  35.48 | 30.48 /  36.29 | 30.18 /  35.17 | 28.52 /  33.36 | 18.45 /  21.57 | 17.28 /  23.08 | 17.76 /  22.64 |
| **No. atoms** | 8482 | 8284 | 8352 | 8211 | 8217 | 8228 | 8262 | 8274 | 8210 | 8358 | 8198 | 8646 |
| **Protein** | 8392 | 7915 | 7945 | 7915 | 7909 | 7907 | 7912 | 7935 | 7931 | 7996 | 7986 | 8392 |
| **Ligands** | 90 | 57 | 57 | 57 | 57 | 57 | 57 | 57 | 33 | 34 | 34 | 90 |
| **Water** | 544 | 284 | 322 | 211 | 223 | 236 | 265 | 254 | 218 | 300 | 150 | 164 |
| ***B*-factors** | 44.82 | 34.34 | 33.25 | 34.64 | 40.97 | 35.09 | 35.52 | 30.69 | 41.84 | 48.25 | 39.94 | 42.16 |
| **Protein** | 44.27 | 34.43 | 33.34 | 34.69 | 41.10 | 35.17 | 35.60 | 30.77 | 41.89 | 48.32 | 39.08 | 42.30 |
| **Ligands** | 36.79 | 26.95 | 32.38 | 29.40 | 32.34 | 28.44 | 31.26 | 24.09 | 28.41 | 32.19 | 21.95 | 36.13 |
| **Water** | 54.51 | 33.47 | 27.06 | 34.37 | 38.62 | 33.88 | 34.30 | 29.50 | 42.22 | 48.88 | 35.76 | 38.15 |
| **Bond lengths (Å)** | 0.008 | 0.001 | 0.001 | 0.002 | 0.002 | 0.001 | 0.001 | 0.004 | 0.001 | 0.003 | 0.001 | 0.002 |
| **Bond angles (**°**)** | 0.885 | 0.414 | 0.424 | 0.455 | 0.436 | 0.409 | 0.426 | 0.673 | 0.422 | 0.670 | 0.417 | 0.469 |
| **Ramachandran favored / allowed / outliers in %** | 98.71 /  1.29 /  0.00 | 96.73 /  3.17 /  0.10 | 97.23 /  2.57 /  0.20 | 96.93 /  2.77 /  0.30 | 96.73 /  2.87 /  0.40 | 97.23 /  2.57 /  0.20 | 96.82 /  2.68 /  0.50 | 96.04 /  3.47  0.50 | 96.13 /  3.57  0.30 | 98.13 /  1.77 /  0.10 | 95.97 /  3.54 /  0.49 | 97.72 /  2.08 /  0.20 |
| **PDB Code** | 7YYQ | 7YYV | 7YYW | 7YYX | 7YYY | 7YYZ | 7YZ0 | 7YZ1 | 7YZ2 | 7YZ3 | 7YZ5 | 7YZ6 |

**Supplementary References:**

1. Hull K, Morstein J, Trauner D. In Vivo Photopharmacology. *Chem Rev* **118**, 10710-10747 (2018).

2. Beharry AA, Woolley GA. Azobenzene photoswitches for biomolecules. *Chem Soc Rev* **40**, 4422-4437 (2011).

3. Borowiak M*, et al.* Photoswitchable Inhibitors of Microtubule Dynamics Optically Control Mitosis and Cell Death. *Cell* **162**, 403-411 (2015).

4. Gaspari R, Prota AE, Bargsten K, Cavalli A, Steinmetz MO. Structural Basis of cis- and trans-Combretastatin Binding to Tubulin. *Chem-Us* **2**, 102-113 (2017).

5. Brouhard GJ, Rice LM. Microtubule dynamics: an interplay of biochemistry and mechanics. *Nat Rev Mol Cell Biol* **19**, 451-463 (2018).

6. Dalbeth N, Lauterio TJ, Wolfe HR. Mechanism of action of colchicine in the treatment of gout. *Clin Ther* **36**, 1465-1479 (2014).

7. Pettit GR, Singh SB, Hamel E, Lin CM, Alberts DS, Garcia-Kendall D. Isolation and structure of the strong cell growth and tubulin inhibitor combretastatin A-4. *Experientia* **45**, 209-211 (1989).

8. Grisham R, Ky B, Tewari KS, Chaplin DJ, Walker J. Clinical trial experience with CA4P anticancer therapy: focus on efficacy, cardiovascular adverse events, and hypertension management. *Gynecol Oncol Res Pract* **5**, 1 (2018).

9. Baytas SN. Recent Advances in Combretastatin A-4 Inspired Inhibitors of Tubulin Polymerization: An Update. *Curr Med Chem* **29**, 3557-3585 (2022).

10. Barnette KG*, et al.* Oral Sabizabulin for High-Risk, Hospitalized Adults with Covid-19: Interim Analysis. *NEJM Evidence* **0**, EVIDoa2200145 (2022).

11. Jerca FA, Jerca VV, Hoogenboom R. Advances and opportunities in the exciting world of azobenzenes. *Nature Reviews Chemistry* **6**, 51-69 (2022).

12. Bozovic O, Jankovic B, Hamm P. Using azobenzene photocontrol to set proteins in motion. *Nature Reviews Chemistry* **6**, 112-124 (2022).

13. Neutze R, Moffat K. Time-resolved structural studies at synchrotrons and X-ray free electron lasers: opportunities and challenges. *Curr Opin Struc Biol* **22**, 651-659 (2012).

14. Tenboer J*, et al.* Time-resolved serial crystallography captures high-resolution intermediates of photoactive yellow protein. *Science* **346**, 1242-1246 (2014).

15. Monteiro DCF, Amoah E, Rogers C, Pearson AR. Using photocaging for fast time-resolved structural biology studies. *Acta Crystallographica Section D* **77**, (2021).

16. Steinmetz MO, Prota AE. Microtubule-Targeting Agents: Strategies To Hijack the Cytoskeleton. *Trends Cell Biol* **28**, 776-792 (2018).

17. Branden G, Neutze R. Advances and challenges in time-resolved macromolecular crystallography. *Science* **373**, 980-+ (2021).

18. Orville AM. Recent results in time resolved serial femtosecond crystallography at XFELs. *Curr Opin Struc Biol* **65**, 193-208 (2020).

19. Gao L*, et al.* A Robust, GFP-Orthogonal Photoswitchable Inhibitor Scaffold Extends Optical Control over the Microtubule Cytoskeleton. *Cell Chem Biol* **28**, 228-241 e226 (2021).

20. Gao L*, et al.* In Vivo Photocontrol of Microtubule Dynamics and Integrity, Migration and Mitosis, by the Potent GFP-Imaging-Compatible Photoswitchable Reagents SBTubA4P and SBTub2M. *J Am Chem Soc* **144**, 5614-5628 (2022).

21. Kirchner S, Pianowski Z. Photopharmacology of Antimitotic Agents. *Int J Mol Sci* **23**, (2022).

22. Pecqueur L*, et al.* A designed ankyrin repeat protein selected to bind to tubulin caps the microtubule plus end. *Proc Natl Acad Sci U S A* **109**, 12011-12016 (2012).

23. La Sala G*, et al.* Structure, Thermodynamics, and Kinetics of Plinabulin Binding to Two Tubulin Isotypes. *Chem-Us* **5**, 2969-2986 (2019).

24. Weinert T*, et al.* Serial millisecond crystallography for routine room-temperature structure determination at synchrotrons. *Nat Commun* **8**, 542 (2017).

25. Weinert T*, et al.* Proton uptake mechanism in bacteriorhodopsin captured by serial synchrotron crystallography. *Science* **365**, 61-65 (2019).

26. Abela R*, et al.* Perspective: Opportunities for ultrafast science at SwissFEL. *Struct Dyn* **4**, 061602 (2017).

27. Wickstrand C*, et al.* A tool for visualizing protein motions in time-resolved crystallography. *Struct Dyn* **7**, 024701 (2020).

28. Skopintsev P*, et al.* Femtosecond-to-millisecond structural changes in a light-driven sodium pump. *Nature*, (2020).

29. Pandey S*, et al.* Time-resolved serial femtosecond crystallography at the European XFEL. *Nature Methods* **17**, 73-+ (2020).

30. Decherchi S, Cavalli A. Thermodynamics and Kinetics of Drug-Target Binding by Molecular Simulation. *Chem Rev* **120**, 12788-12833 (2020).

31. Prota AE*, et al.* The Novel Microtubule-Destabilizing Drug BAL27862 Binds to the Colchicine Site of Tubulin with Distinct Effects on Microtubule Organization. *Journal of Molecular Biology* **426**, 1848-1860 (2014).

32. Dorleans A, Gigant B, Ravelli RBG, Mailliet P, Mikol V, Knossow M. Variations in the colchicine-binding domain provide insight into the structural switch of tubulin. *P Natl Acad Sci USA* **106**, 13775-13779 (2009).

33. Ravelli RBG*, et al.* Insight into tubulin regulation from a complex with colchicine and a stathmin-like domain. *Nature* **428**, 198-202 (2004).

34. Muhlethaler T, Gioia D, Prota AE, Sharpe ME, Cavalli A, Steinmetz MO. Comprehensive Analysis of Binding Sites in Tubulin. *Angew Chem Int Ed Engl* **60**, 13331-13342 (2021).

35. Knossow M, Campanacci V, Khodja LA, Gigant B. The Mechanism of Tubulin Assembly into Microtubules: Insights from Structural Studies. *iScience* **23**, 101511 (2020).

36. Brouhard GJ, Rice LM. The contribution of alphabeta-tubulin curvature to microtubule dynamics. *J Cell Biol* **207**, 323-334 (2014).

37. Gaillard N*, et al.* Inhibiting parasite proliferation using a rationally designed anti-tubulin agent. *Embo Mol Med* **13**, (2021).

38. Mehrabi P*, et al.* Time-resolved crystallography reveals allosteric communication aligned with molecular breathing. *Science* **365**, 1167-1170 (2019).

39. Pandey S*, et al.* Observation of substrate diffusion and ligand binding in enzyme crystals using high-repetition-rate mix-and-inject serial crystallography. *Iucrj* **8**, 878-895 (2021).

40. Butryn A*, et al.* An on-demand, drop-on-drop method for studying enzyme catalysis by serial crystallography. *Nat Commun* **12**, 4461 (2021).

41. Schmidt M. Reaction Initiation in Enzyme Crystals by Diffusion of Substrate. *Crystals* **10**, (2020).

42. Ricart-Ortega M, Font J, Llebaria A. GPCR photopharmacology. *Mol Cell Endocrinol* **488**, 36-51 (2019).

43. Velema WA, Szymanski W, Feringa BL. Photopharmacology: beyond proof of principle. *J Am Chem Soc* **136**, 2178-2191 (2014).

44. Leippe P, Frank JA. Designing azobenzene-based tools for controlling neurotransmission. *Curr Opin Struct Biol* **57**, 23-30 (2019).

45. Weierstall U*, et al.* Lipidic cubic phase injector facilitates membrane protein serial femtosecond crystallography. *Nat Commun* **5**, 3309 (2014).

46. Nogly P*, et al.* Lipidic cubic phase injector is a viable crystal delivery system for time-resolved serial crystallography. *Nat Commun* **7**, 12314 (2016).

47. James D*, et al.* Improving High Viscosity Extrusion of Microcrystals for Time-resolved Serial Femtosecond Crystallography at X-ray Lasers. *J Vis Exp*, (2019).

48. Schulz EC*, et al.* The hit-and-return system enables efficient time-resolved serial synchrotron crystallography. *Nat Methods* **15**, 901-904 (2018).

49. Fuller FD*, et al.* Drop-on-demand sample delivery for studying biocatalysts in action at X-ray free-electron lasers. *Nat Methods* **14**, 443-449 (2017).

50. Borowiak M*, et al.* Optical Manipulation of F-Actin with Photoswitchable Small Molecules. *J Am Chem Soc* **142**, 9240-9249 (2020).

51. Pospich S*, et al.* Cryo-EM Resolves Molecular Recognition Of An Optojasp Photoswitch Bound To Actin Filaments In Both Switch States. *Angew Chem Int Edit* **60**, 8678-8682 (2021).

52. Wang H, Robinson RC, Burtnick LD. The structure of native G-actin. *Cytoskeleton (Hoboken)* **67**, 456-465 (2010).

53. Westphal MV*, et al.* Synthesis of Photoswitchable Delta(9)-Tetrahydrocannabinol Derivatives Enables Optical Control of Cannabinoid Receptor 1 Signaling. *J Am Chem Soc* **139**, 18206-18212 (2017).

54. Hua T*, et al.* Crystal structures of agonist-bound human cannabinoid receptor CB1. *Nature* **547**, 468-471 (2017).

55. Arkhipova V*, et al.* Structural Aspects of Photopharmacology: Insight into the Binding of Photoswitchable and Photocaged Inhibitors to the Glutamate Transporter Homologue. *Journal of the American Chemical Society* **143**, 1513-1520 (2021).

56. Reynders M*, et al.* Controlling the Covalent Reactivity of a Kinase Inhibitor with Light. *Angew Chem Int Ed Engl* **60**, 20178-20183 (2021).

57. Muhlethaler T*, et al.* Crystallization Systems for the High-Resolution Structural Analysis of Tubulin-Ligand Complexes. *Methods Mol Biol* **2430**, 349-374 (2022).

58. Sugahara M*, et al.* Hydroxyethyl cellulose matrix applied to serial crystallography. *Sci Rep* **7**, 703 (2017).

59. Nass K*, et al.* Pink-beam serial femtosecond crystallography for accurate structure-factor determination at an X-ray free-electron laser. *IUCrJ* **8**, (2021).

60. Zeldin OB, Gerstel M, Garman EF. RADDOSE-3D: time- and space-resolved modelling of dose in macromolecular crystallography. *Journal of Applied Crystallography* **46**, 1225-1230 (2013).

61. White TA*, et al.* CrystFEL: a software suite for snapshot serial crystallography. *Journal of Applied Crystallography* **45**, 335-341 (2012).

62. White TA*, et al.* Recent developments in CrystFEL. *J Appl Crystallogr* **49**, 680-689 (2016).

63. Gevorkov Y*, et al.* XGANDALF - extended gradient descent algorithm for lattice finding. *Acta Crystallogr A Found Adv* **75**, 694-704 (2019).

64. Tickle IJ, Flensburg, C., Keller, P., Paciorek, W., Sharff, A., Vonrhein, C., Bricogne, G. . STARANISO.). Cambridge, United Kingdom: Global Phasing Ltd. (2018).

65. Adams PD*, et al.* PHENIX: building new software for automated crystallographic structure determination. *Acta Crystallogr D Biol Crystallogr* **58**, 1948-1954 (2002).

66. Winn MD*, et al.* Overview of the CCP4 suite and current developments. *Acta Crystallogr D Biol Crystallogr* **67**, 235-242 (2011).

67. Gorel A, Schlichting I, Barends TRM. Discerning best practices in XFEL-based biological crystallography - standards for nonstandard experiments. *Iucrj* **8**, 532-543 (2021).

68. Nogly P*, et al.* Retinal isomerization in bacteriorhodopsin captured by a femtosecond x-ray laser. *Science* **361**, (2018).

69. Emsley P, Cowtan K. Coot: model-building tools for molecular graphics. *Acta Crystallogr D Biol Crystallogr* **60**, 2126-2132 (2004).

70. Bricogne G BE, Brandl M, Flensburg C, Keller P, Paciorek W, Roversi P, Sharff A, Smart O, Vonrhein C, Womack T BUSTER version 2.10.4.). Cambridge, United Kingdom: Global Phasing Ltd. (2021).

71. Salentin S, Schreiber S, Haupt VJ, Adasme MF, Schroeder M. PLIP: fully automated protein-ligand interaction profiler. *Nucleic Acids Res* **43**, W443-447 (2015).

72. Smith RHB, Dar AC, Schlessinger A. PyVOL: a PyMOL plugin for visualization, comparison, and volume calculation of drug-binding sites. *bioRxiv*, 816702 (2019).

73. Chovancova E*, et al.* CAVER 3.0: a tool for the analysis of transport pathways in dynamic protein structures. *PLoS Comput Biol* **8**, e1002708 (2012).

74. Slavov C*, et al.* The ultrafast reactions in the photochromic cycle of water-soluble fulgimide photoswitches. *Phys Chem Chem Phys* **18**, 10289-10296 (2016).

75. Jacobson MP, Friesner RA, Xiang Z, Honig B. On the role of the crystal environment in determining protein side-chain conformations. *J Mol Biol* **320**, 597-608 (2002).

76. Sastry GM, Adzhigirey M, Day T, Annabhimoju R, Sherman W. Protein and ligand preparation: parameters, protocols, and influence on virtual screening enrichments. *J Comput Aided Mol Des* **27**, 221-234 (2013).

77. D.A. Case HMA, K. Belfon, I.Y. Ben-Shalom, S.R. Brozell, D.S. Cerutti, T.E. Cheatham, III, G.A. Cisneros, V.W.D. Cruzeiro, T.A. Darden, R.E. Duke, G. Giambasu, M.K. Gilson, H. Gohlke, A.W. Goetz, R. Harris, S. Izadi, S.A. Izmailov, C. Jin, K. Kasavajhala, M.C. Kaymak, E. King, A. Kovalenko, T. Kurtzman, T.S. Lee, S. LeGrand, P. Li, C. Lin, J. Liu, T. Luchko, R. Luo, M. Machado, V. Man, M. Manathunga, K.M. Merz, Y. Miao, O. Mikhailovskii, G. Monard, H. Nguyen, K.A. O’Hearn, A. Onufriev, F. Pan, S. Pantano, R. Qi, A. Rahnamoun, D.R. Roe, A. Roitberg, C. Sagui, S. Schott-Verdugo, J. Shen, C.L. Simmerling, N.R. Skrynnikov, J. Smith, J. Swails, R.C. Walker, J. Wang, H. Wei, R.M. Wolf, X. Wu, Y. Xue, D.M. York, S. Zhao, and P.A. Kollman. Amber 21. In: *University of California, San Francisco*) (2021).

78. Jorgensen WL, Chandrasekhar J, Madura JD, Impey RW, Klein ML. Comparison of Simple Potential Functions for Simulating Liquid Water. *J Chem Phys* **79**, 926-935 (1983).

79. Maier JA, Martinez C, Kasavajhala K, Wickstrom L, Hauser KE, Simmerling C. ff14SB: Improving the Accuracy of Protein Side Chain and Backbone Parameters from ff99SB. *J Chem Theory Comput* **11**, 3696-3713 (2015).

80. Allner O, Nilsson L, Villa A. Magnesium Ion-Water Coordination and Exchange in Biomolecular Simulations. *J Chem Theory Comput* **8**, 1493-1502 (2012).

81. Meagher KL, Redman LT, Carlson HA. Development of polyphosphate parameters for use with the AMBER force field. *J Comput Chem* **24**, 1016-1025 (2003).

82. Salomon-Ferrer R, Gotz AW, Poole D, Le Grand S, Walker RC. Routine Microsecond Molecular Dynamics Simulations with AMBER on GPUs. 2. Explicit Solvent Particle Mesh Ewald. *Journal of Chemical Theory and Computation* **9**, 3878-3888 (2013).

83. Barducci A, Bussi G, Parrinello M. Well-tempered metadynamics: A smoothly converging and tunable free-energy method. *Phys Rev Lett* **100**, (2008).

84. Van der Spoel D, Lindahl E, Hess B, Groenhof G, Mark AE, Berendsen HJC. GROMACS: Fast, flexible, and free. *J Comput Chem* **26**, 1701-1718 (2005).

85. Tribello GA, Bonomi M, Branduardi D, Camilloni C, Bussi G. PLUMED 2: New feathers for an old bird. *Comput Phys Commun* **185**, 604-613 (2014).
