# Supplementary material for: Watching the release of a photopharmacological drug from tubulin using time-resolved serial crystallography
Source: Nat Commun. 2023 Feb 17;14:903. doi: 10.1038/s41467-023-36481-5 (PMC9936131; doi:10.1038/s41467-023-36481-5)
Supplement: Supplementary file 3 — Description of Additional Supplementary Files [file 41467_2023_36481_MOESM3_ESM.docx]

Description of Additional Supplementary

**The additional supplementary includes:**

**Supplementary coordinate files**: Start (Dataset 1) and end (Dataset 2) points of the molecular dynamic simulation shown in Figure 3D.

**Supplementary Movie 1: Evolution of difference electron density along the azo-CA4 ligand over time**. The movie shows the overlay of dark (grey) and time-resolved (tubulin in blue, azoCA4 in orange) structures with plotted electron difference densities (F_obs_(light)-F_obs_(dark), negative in red and positive in green). The contour level was set to 3 σ for most time delays. For better comparison it is shown to higher levels for the ~100 ms time delay from the synchrotron experiment where we achieved higher activation levels.

**Supplementary Movie 2: Movie illustrating the principal steps upon azo-CA4 release.** The movie is prepared from morphs between the nine molecular snapshots and highlights the effect of azo-CA4 (before and after illumination as yellow and orange sticks, respectively) isomerization, relaxation, release and the resulting collapse of the colchicine binding site (α-tubulin in blue and β-tubulin in cyan and βT7 loop in green, respectively, cartoon or surface representations, selected residues are shown in stick representation).
